# A review of real-world evidence on preemptive pharmacogenomic testing for preventing adverse drug reactions: a reality for future health care

**DOI:** 10.1038/s41397-024-00326-1

**Published:** 2024-03-15

**Authors:** Santenna Chenchula, Shubham Atal, Chakradhara Rao S Uppugunduri

**Affiliations:** 1 Department of Pharmacology, All India Institute of Medical Sciences (AIIMS), Bhopal, India; 2 CANSEARCH Research Platform in Pediatric Oncology and Hematology, Department of Pediatrics, Gynecology and Obstetrics, University of Geneva, Geneva, Switzerland

**Keywords:** Disease prevention, Diagnosis

## Abstract

Adverse drug reactions (ADRs) are a significant public health concern and a leading cause of hospitalization; they are estimated to be the fourth leading cause of death and increasing healthcare costs worldwide. Carrying a genetic variant could alter the efficacy and increase the risk of ADRs associated with a drug in a target population for commonly prescribed drugs. The use of pre-emptive pharmacogenetic/omic (PGx) testing can improve drug therapeutic efficacy, safety, and compliance by guiding the selection of drugs and/or dosages. In the present narrative review, we examined the current evidence of pre-emptive PGx testing-based treatment for the prevention of ADRs incidence and hospitalization or emergency department visits due to serious ADRs, thus improving patient safety. We then shared our perspective on the importance of preemptive PGx testing in clinical practice for the safe use of medicines and decreasing healthcare costs.

## Introduction

Adverse drug reactions (ADRs) are a significant public health concern and a leading cause of hospitalization and mortality worldwide in both developed and developing countries [1–6]. ADRs account for 3–6% of hospital admissions in the United States, 2.5–10.6% of admissions in Europe, and 134 million adverse events occur annually in low- and middle-income countries (LMICs) due to unsafe care in hospitals, resulting in 2.6 million deaths [1–3, 7–11]. The costs of treating ADRs in a hospital setting vary between different units, with estimates of $13,994 in a nonintensive care unit (ICU) and $19,685 in an ICU setting [10–13]. Furthermore, ADRs are also associated with decreased patient compliance with treatment, leading to a substantial worsening of the disease, mortality and increased healthcare costs [12]. The potential to reduce morbidity and mortality through increased patient safety, fewer ADRs, and cost savings due to improved drug efficacy is immense [14, 15].

Although many ADRs are preventable and often attributed to human error, others appear to be idiosyncratic and potentially influenced by genetic factors [16–18]. Almost 50% of spontaneously reported ADRs may have identifiable causes, most likely explained by genetic variability [19, 20]. The genetic predisposition to ADR is increasingly known/investigated, particularly for anticancer, cardiovascular and neuropsychiatric therapeutics [19]. Several drug-specific severe idiosyncratic adverse effects, including severe hemolysis with glucose-6-phosphate dehydrogenase (G6PD) deficiency, malignant hyperthermia, epidermal tissue necrosis (Lyell’s syndrome and Stevens-Johnson syndrome), drug reactions with eosinophilia and systemic symptoms (DRESS), thyroid diseases, porphyria, aplastic anemia, long QT syndrome, and Brugada syndrome, are now explained by genetic predisposition [21–23].

Currently, the most common method for preventing ADRs due to prescribing drugs is the trial-and-error approach [24]. Four out of five patients are likely to carry a genetic variant that could alter the efficacy of commonly prescribed drugs [4]. Optimizing drug prescribing decisions based on patient genetic data may help reduce ADRs and improve drug effectiveness [24]. Pharmacogenomics (PGx) refers to the influence of various components of the genome on drug response, while pharmacogenetics (PGx) is a subcategory of pharmacogenomics that focuses on the role of genetic variation in drug targets, transporters and metabolizing enzymes and is known to predict some of the variability in drug effectiveness and safety [24]. PGx variants strongly affect drug disposition or metabolism, significantly contributing to the adverse outcomes associated with therapies [24]. At least one drug with a clinical annotation in the Pharmacogenomics Knowledge Base (PharmGKB) was responsible for 30% of the ADRs upon hospital admission, suggesting that some of these ADRs could have been predicted through PGx testing [10, 11]. Preemptive PGx testing refers to the practice of testing an individual’s genetic makeup before prescribing any drugs that guide the selection of drugs and dosages that are most likely to be effective and well tolerated [24, 25]. The use of PGx can optimize drug therapy by identifying patients at risk of potential drug interactions and adverse events and guiding the selection of drugs and dosages that are most likely to be effective and well tolerated [24, 25]. Therefore, this narrative review aims to explore the current evidence on preemptive pharmacogenomic testing in healthcare for the prevention of ADRs and to discuss the gaps in the literature that need to be addressed to strengthen the implementation of such testing in healthcare settings.

## Methods

**Fig. 1:**
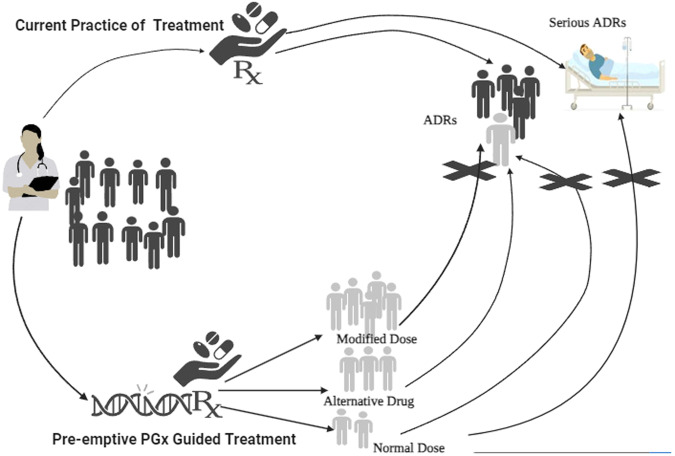
Effect of preemptive pharmacogenomic test-based treatment versus current practice of treatment in preventing adverse drug reactions. Dark shared individuals receiving treatment without preemptive genetic testing are at higher risk of developing ADRs, compared to the individuals (light shaded) with prior PGx information that can guide alterations in the dose / drug avoiding this risk of developing ADRs.

We examined the literature from inception to July 2023, in Medline, Google Scholar, and Cochrane library using the Medical Subject Headings (MeSH) terms and keywords: Adverse drug reactions; Adverse reactions OR Adverse drug events; Pharmacogenetic testing; pharmacogenomic testing; preemptive pharmacogenetic*; preemptive pharmacogenomic*. No restrictions on language were applied. The primary outcome of the present review was to provide an informed perspective on the potential of preemptive PGx testing for improving treatment outcomes by preventing ADRs and reducing healthcare costs by minimizing the occurrence of ADRs, severity and hospitalization associated with serious ADRs over usual treatment (Fig. 1) (Created using BioRender.com).

## Results

### Current evidence on preemptive PGx testing in healthcare for preventing ADRs and ensuring safe use of medicines

The findings of the Preemptive Pharmacogenomic Testing for Preventing Adverse Drug Reactions (PREPARE) trial, conducted by the ubiquitous pharmacogenomics consortium, drove us toward the goal of implementing PGx in clinical practice [22]. This study offers robust evidence supporting the use of preemptive PGx testing to prevent ADRs and underscores the importance of integrating genetic testing into routine clinical practice. A total of 6944 patients from primary care, oncology, and general medicine units were enrolled and randomly assigned to two groups: genotype-guided drug treatment (n = 3342) and standard care (n = 3602). Overall, 10,718 ADRs were reported in 3303 patients. The PGx panel included genes responsible for drug metabolism, transport, and receptor activity and was designed to predict the risk of ADRs associated with 56 commonly used medications. The study investigators followed the Dutch Pharmacogenetics Working Group (DPWG) guidelines to examine variants in 12 genes, including *CYP2B6, CYP2C9, CYP2C19, CYP2D6, CYP3A5, DPYD, F5, HLA-B, SLCO1B1, TPMT, UGT1A1*, and *VKORC1*. Patients who underwent testing received personalized medication plans, including recommendations for drug dosages and/or alternative medications, based on their genetic profile. Among the patients, 93.5% had at least one actionable gene variant. The trial results demonstrated a significant reduction in the incidence of ADRs associated with PGx testing. Patients who underwent testing had a 33% lower risk of experiencing ADRs than did those receiving standard care (21.5% vs. 28.6%) [22]. The reduction in ADRs was particularly notable for patients taking medications with a high risk of ADRs, which involved 39 drugs. These findings align with those of the Vanderbilt PREDICT study and the Mayo-Baylor RIGHT 10 K study, where PGx testing was found to be relevant for the majority of the studied population [23, 24].

The Pharmacogenomic Resource for Enhanced Decisions in Care and Treatment (PREDICT) program was launched in 2010 by Vanderbilt University Medical Center in the USA [23]. The initial platform used was the VeraCode ADME core panel, which tested 184 variants and 34 genes. The study findings indicate that PGx testing has yielded significant outcomes in the Vanderbilt PREDICT program and related initiatives. Among the first 10,000 patients tested, the frequency of highly actionable genetic variants varied across different drug–gene pairs, ranging from 0% to 2.5%. Overall, 91% of the subjects had at least one variant in the tested drug–gene pairs, emphasizing the prevalence of pharmacogenetic variations. These data highlight the advantages of a preemptive approach, where genetic information is available at the point of care. Additionally, the study demonstrated that implementing a multiplexed strategy in the preemptive approach can reduce genotyping costs [23].

The “RIGHT 10 K” study was a large-scale PGx testing program conducted by researchers at the Mayo Clinic and Baylor College of Medicine in the USA [24]. This study aimed to evaluate the clinical utility of incorporating PGx testing in routine clinical care. The study evaluated the impact of preemptive PGx testing and optimized the workflow in the clinical setting using an 84-gene next-generation sequencing panel, which included *SLCO1B1, CYP2C19, CYP2C9*, and *VKORC1*, along with a custom-designed *CYP2D6* testing cascade to genotype the 1013 subjects in laboratories approved by the Clinical Laboratory Improvement Act. The percentage of patients carrying actionable PGx variants ranged from 30% (*SLCO1B1*) to 79% (*CYP2D6*). When considering all five genes together, 99% of the subjects carried actionable PGx variants in at least one gene [24].

In another prospective, open-label, randomized controlled trial that evaluated the clinical impact of PGx profiling, Elliott et al. reported that preemptive PGx testing of six genes led to a reduction in the number of rehospitalizations, emergency department (ED) visits, and the composite number of rehospitalizations plus ED visits at 60 days by 52%, 42%, and 48%, respectively [26]. The study also showed that patients who received preemptive testing had a 52% reduction in ADRs compared to those who did not [26].

Reports from special populations also indicated the substantial benefit of PGx testing compared to untested groups [27, 28]. Similarly, Brixner et al. reported lower hospitalization rates in the elderly population (9.8%) when PGx variants were tested than in the untested group (16.1%) [27]. This particular study compared healthcare resource utilization (HRU) and clinical decision-making for elderly patients based on cytochrome P450 (CYP) PGx testing and the use of a comprehensive medication management clinical decision support tool (CDST) in comparison to a cohort of similar patients but not tested for PGx variants [27]. The ED visit rate in this study was 4.4% in the PGx-tested group, compared to 15.4% in the untested group [27]. Overall, the rate of HRU was 72.2% in the PGx-tested group and 49.0% in the untested group, and the estimated mean cost savings were $218 in the tested group relative to the untested group [27].

Similarly, the PGEN4Kids study (PG4KDS) by St. Jude Children’s Research Hospital, with the objective of preemptive PGx testing (~300 genes) in the pediatric population (n = 1559), revealed that 78% of them had at least one actionable high-risk genotype in the *TPMT, CYP2D6, SLCO1B1* and *CYP2C19* populations that could affect their high-risk drug (12 molecules) therapy [28]. Preemptive testing can be implemented by either preemptive candidate gene testing specifically or extracting candidate gene information from whole-exome or whole-genome sequencing [28]. Another study by Fagerness et al. assessed the outcomes of PGx testing in a pediatric tertiary care setting. This study implemented a point-of-care model for targeted gene‒drug pairs (n = 57) and a preemptive model informed by whole-genome sequencing (n = 115) that evaluated a broad range of drugs [29]. It is also known that the genotypes of 36.8% of children were incompatible with standard treatment regimens, while 80.0% of children were recommended to receive nonstandard treatment regimens based on their six-gene PGx profile [29]. Other relevant benefits such as medication change, increased medication adherence rates, and cost savings with preemptive PGx testing is also well known [30, 31]. Evidence on the utility of the PGx test has also resulted in regulatory guidelines such as the implementation of *DPYD* testing by EMEA and other PGx testing guidelines by the FDA, which have been shown to reduce the costs, severe toxicities and hospitalizations of patients receiving treatment with fluoropyrimidines [32–34].

A systematic review of the current evidence on the impact of PGx testing on hospital admissions and medication changes compared to that of participants who received treatment as usual (TAU) demonstrated that medication changes occurred significantly more frequently in the PGx-tested group across 4 out of 5 studies. Furthermore, all-cause hospitalization occurred less frequently in the PGx-tested group than in the TAU group [35].

A 12-week, double-blind, parallel, multicenter randomized controlled trial by Pérez et al. in 316 adult patients with major depressive disorder (MDD) evaluated the effectiveness of preemptive PGx testing-guided therapy over treatment as usual (TAU) and revealed that, in addition to significant improvement in treatment response, the burden of side effects was significantly reduced at 12 weeks in the PGx-guided treatment group [36]. In another multicentre randomized clinical trial in the Netherlands, Vos, Cornelis et al. compared preemptive PGx informed treatment (PIT) with usual treatment among 111 patients with depressive disorders and reported that patients in the PIT group experienced fewer severe adverse effects than patients in the usual treatment group with faster attainment of therapeutic plasma concentrations [37].

The utility of PGx testing in routine clinical practice is evident from multiple reports from different geographical regions, with varying impacts defined by the variant allele frequencies in candidate genes among specific ethnicities [38, 39]. Huang et al. screened a total of 22,918 participants from 20 provinces in China to analyse the variant allele frequencies of 15 pharmacogenes of 31 drugs based on preemptive PGx testing guidelines established by the Clinical Pharmacogenomics Implementation Consortium (CPIC) [40]. This study demonstrated that a total of 20 drugs have a higher risk for ADRs, indicating genotype–ADR associations [40]. Furthermore, a naturalistic, unblinded trial investigating the effects of preemptive PGx testing-based treatment among 685 psychiatric patients showed that at the end of 3 months, patients reported a significant decrease in medication side effects (P < 0.001) [41]. In another study, Deenen et al. investigated the safety of DPYD*2 A genotype-guided treatment in 2038 patients and reported that proactive *DPYD* genotyping and personalized dosing substantially decreased the incidence of fluoropyrimidine-induced toxicity in comparison to that in historical controls. The risk plummeted from 73% to 28%, and the occurrence of drug-induced fatalities decreased from 10% to 0% [42]. Furthermore, the findings of the Medco-Mayo Warfarin Effectiveness study (MM-WES) demonstrated that preemptive genotyping of the *CYP2C9* and *VKORC1* genes resulted in a 43% reduction in the risk of hospitalization due to bleeding or thromboembolism, with an overall 31% decrease in hospitalizations compared to those in the control group [43]. Another randomized clinical Genetic Informatics Trial (GIFT) study focusing on warfarin among 1650 patients demonstrated that patients receiving genotype-guided therapy had a significantly decreased combined risk of major bleeding, having an international normalized ratio (INR) of 4 or greater, venous thromboembolism or death [44]. Another study from South Asia (India) reported the presence of approximately 134 potentially deleterious PGx variants at a frequency of more than 10%, which may affect the function of 102 pharmacogenes that are associated with drug response and ADRs [45]. This particular study also highlighted that, on average, each individual of Indian origin may carry eight PGx variants impacting drug dose or choice of treatment [45].

Several reports are emerging from other geographical regions and ethnicities highlighting the utility or potential of clinical management using preemptive PGx testing [46–50]. However, geographic variations were observed, highlighting the development of region-specific PGx testing panels.

## Discussion

Preventing ADRs and ensuring the safe use of drugs are the major goals in clinical practice, and PGx testing has been proposed as a potential strategy for achieving these goals [15, 51–55]. Current evidence indicates the potential utility of preemptive PGx testing in healthcare, especially for improving patient safety. Several interesting real-world studies have supported the role of PGx in preventing ADRs associated with medication. It has been observed that more than 100 ADRs can be prevented in patients with cancer who are treated with PGx actionable medications [15].

A few examples of preemptive PGx testing of genetic variants associated with ADRs include HLA-B*57:01 for abacavir, HLA-B*15:02 for phenytoin, fosphenytoin, HLA-B*15:02 and HLA-A*31:01 for carbamazepine; HLA-B*15:02 and HLA-A*24:02 for lamotrigine; HLA-B*58:01 for allopurinol; *CYP2C19* for clopidogrel; *TPMT*, *NUDT15* for 6-mercaptopurine; azathioprine; and cisplatin; *DPYD* for fluoropyrimidines; *CYP2C9* and *VKORC1* for coumarin derivatives; *MTHFR* for methotrexate treatment; factor V Leiden for oral contraception; and *CYP2D6, CYP2C19, CYP2C9* and *CYP2B6* for neuropsychiatric drug prescription[15, 50–56]. All of the examples are enlisted to emphasize utility coverage in various domains of clinical care.

Findings from well-conducted studies have suggested important implications of PGx testing for clinical practice and healthcare policy, as personalized medicine becomes increasingly important in providing high-quality, safe, and effective healthcare. Accumulating evidence suggests that PGx accounts for a wide range (20-95%) of drug response variability, significantly impacting the incidence and severity of ADRs [22–24, 26–29]. Approximately 50% of currently used drugs already have an identified PGx profile, which is useful for preemptive genotyping and offers clinical benefits to patients by improving efficacy and reducing ADRs [57–60].

Overall, PGx testing has the potential to optimize drug therapy by identifying clinically significant ADRs and potential drug interactions. This can lead to a reduction in ED visits and hospitalizations associated with serious adverse events, ultimately decreasing healthcare costs [61]. Major evidence from three large RCTs and other real-world studies included in the present study is insufficient for implementing preemptive PGx for the safe use of medications in other geographical settings. Hence, further randomized controlled trials (RCTs) are warranted in limited resource settings to assess the cost-effectiveness of preemptive PGx in preventing ADRs, reducing healthcare resource utilization, and improving long-term patient care. Additionally, genome-wide association studies (GWASs) are needed to identify optimal pharmacokinetic and pharmacodynamic genes for predicting patient response and the risk of ADRs, particularly in therapeutic areas such as cancer chemotherapeutic agents, cardiovascular medications, and neuropsychiatric drugs. Several international associations and organizations, such as the United States Food and Drug Administration (FDA), Clinical Pharmacogenetics Implementation Consortium (CPIC), and Dutch Pharmacogenetics Working Group (DPWG), provide evidence-based guidelines for the safe and effective use of drugs based on genetic testing results [60]. The FDA has already implemented PGx information on the labels of approximately 200 medications for safety monitoring [58]. Recent draft guidance from the National Institute for Health and Care Excellence (NICE) recommends *CYP2C19* genotype testing for people at risk of a secondary stroke [61].

Despite the increasing number of PGx studies, the use of PGx in clinical practice has been very slow due to various challenges, especially in developing countries. These challenges include limited randomized trials demonstrating improved clinical outcomes based on genotype, methodological limitations in published studies, turnaround times and availability of genotyping tests, regulatory and ethical concerns, lack of cost-effectiveness analyses, lack of education and training for health care providers, potential delays in therapy while awaiting test results, and the need for patient privacy and confidentiality [55–57, 62]. As more evidence emerges and testing techniques advance, the cost of testing is expected to decrease, increasing accessibility [55–57]. A comprehensive approach involving collaboration between healthcare professionals, regulatory bodies, and patients is necessary to promote the appropriate use of preemptive PGx testing and develop guidelines and policies [55–57].

## Conclusion

In conclusion, preemptive PGx testing holds promising potential for predicting and preventing ADRs, thus reducing healthcare resource utilization and improving long-term patient care. Major evidence from several RCTs included in the present literature review is sufficient to strengthen the existing evidence on the use of preemptive PGx for preventing ADRs and safely using medications. Further research is necessary in developing countries to assess the effectiveness of preemptive PGx testing for preventing ADRs, reducing healthcare resource utilization, and improving long-term patient care. Additionally, GWASs are warranted to identify optimal pharmacokinetic and pharmacodynamic genes for predicting patient response and the risk of ADRs, across all therapeutic areas.
